# Supplementary material for: Ergodicity breaking transition in a glassy soft sphere system at small but non-zero temperatures
Source: Sci Rep. 2018 Jan 30;8:1837. doi: 10.1038/s41598-018-20152-3 (PMC5789873; doi:10.1038/s41598-018-20152-3)
Supplement: Supplementary file 1 — Supplementary Notes [file 41598_2018_20152_MOESM1_ESM.pdf]

Supplementary Notes on "Ergodicity breaking transition in a glassy soft sphere system at small but non-zero temperatures"

Moumita Maiti[1] **and Michael Schmiedeberg**[1,*]
[1]Institut für Theoretische Physik 1, Friedrich-Alexander-Universität Erlangen-Nürnberg, Staudtstr. 7, 91058 Erlangen, Germany
[*]michael.schmiedeberg@fau.de

Supplementary Note 1:
Modifications of the protocol

In this section we present different models, where we vary the protocol that is employed to access the ergodicity breaking transition in the limit of rare barrier crossing events. In Fig. 1(a) we summarize the ranges that are obtained for the transition packing fraction. The curves for the fraction of overlaps as a function of step number for cases close to this transition are displayed in Fig. 1(b,c,d,e). The employed models are

- Model A: The model that is used in the main text and that is explained in detail therein.
- Model B: In this variation of model A we only allow overlapping particles to take part in the protocol. Therefore, touching particles can no longer be randomly displaced.
- Model C: Variation of model A, where in a random step particles are only displaced if in the chosen direction there is a crossing of an energy barrier. Otherwise the particles is not moved. Therefore, minimization only takes place during minimization steps.
- Model D: The minimization is done according to the steepest descent algorithm. Otherwise the protocol of model A is used. Note that minimization using the steepest descend algorithm usually is less efficient than the conjugate gradient algorithm that is used in models A to C. As a consequence, the relaxation just below the transition is very slow and noisy as can be seen for the orange curve for $\phi = 0.53$ in Fig. 1(d). As a consequence, in order to differentiate between relaxing and non relaxing systems close to the transition, we also analyzed the structure. In case of a persisting percolated cluster we are sure to be in the non-ergodic phase, while below the transition the system disassociates into distinct non-percolating clusters of overlapping particles that slowly shrink.
- Model E: In this model completely different protocols for minimization and random steps are used. The minimization steps corresponds to a deterministic athermal jamming step as used in[S1]. In such a step two overlapping particles are deterministically displaced along the line given by the two center points of the particles until the particles are no longer overlapping but only touching. In random steps that occur with probability p the touching particles in addition are rotated around the center of mass by an random angle. More details are given in[S1], where we studied the individual protocols but not their mixing. Note that the minimization in this model is very ineffective such that no large systems can be

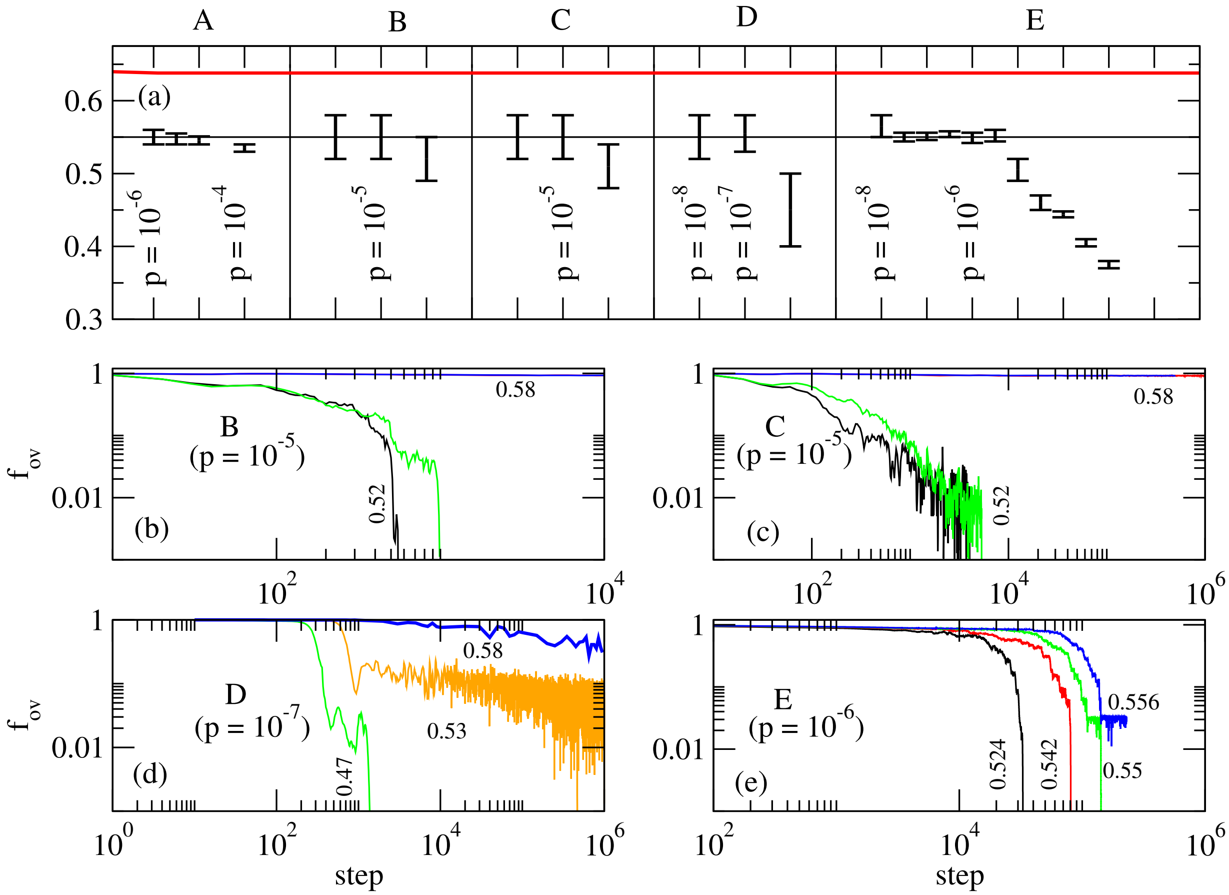

Supplementary Figure 1: Modifications of the protocol. (a) Ranges of the transition packing fraction for various models with different protocols. The models are labeled with the capital letters displayed on top of the figure and explained in the main text. The black horizontal line marks the packing fraction 0.55, the red horizontal line denotes the packing fraction of athermal jamming. (b-e) Fraction of overlapping particles f_{ov} as a function of step number t for some of the cases for the models shown in (a). The respective models are marked by the capital letters in the figures. The numbers denote the packing fractions. The employed system sizes are the following: (b,c) $N = 10^5$ (black and red lines) and $N = 10^6$ (green and blue lines), (d) $N = 10^6$, (e) average over 25 runs with $N = 25000$ particles.

studied within a reasonable computation time. For the shown results, we averaged over 25 runs with $N = 25000$ particles. As a consequence, the drop after a plateau has been reached for packing fraction 0.55 in Fig. 1(e) probably is artifact due to the small system size. Therefore, in principle it is possible that the error bars shown in Fig. 1(a) for model E differ from error bars obtained in larger systems. However, up to the system size that we could consider, even in this completely different model the plateaus start to develop at the same packing fraction as for the other models.

Concerning finite size effects, we want to point out that slower minimization protocols might require to go to smaller values of p in order to reach the limit where the transition packing fraction no longer depends on p. As a consequence, such protocols might require larger system sizes to obtain a comparable accuracy for the transition packing fraction. In general, the width of the range for the transition packing fraction is mainly limited due to finite size effects. In model A we performed an extensive finite size analysis (cf. Supplementary Note 2) and as a consequence can narrow the range significantly. For models B and C we checked two different system size to show that the given range for the transition packing fraction is not affected by the system size. In case of model E we have to point out that significant finite size effects are still present. Especially the result for $p = 10^{-8}$ is probably strongly affected by the system size.

In summary, in the limit of rare barrier crossing events, all models lead to a range for the transition packing fraction that is in agreement with the transition packing fraction determined by model A in the main text.

Supplementary Note 2:
Analysis of system size effects

In the following we show how the relaxation process might be affected by finite size effects. Furthermore, at the end of this section we explain how we choose our system sizes such that the transitions that we determine do not depend on these sizes.

Fig. 2(a) demonstrates that below the transition packing fraction smaller systems relax sooner than larger systems. For the small probability $p = 10^{-5}$ we point out to another interesting observation: We find that the relaxation obeys an unexpected power law with an exponent -1.5. Note that this power law only occurs below the transition and is not related to the critical power law decay t^{α} with $\alpha = -0.732$ which occurs directly at the transition.

For the relaxation above the transition, we depict different system sizes for $p = 0.1$ in Fig. 2(b) and for $p = 10^{-4}$ in Fig. 2(c). For small systems the fraction of overlapping particles might suddenly decay to zero instead of approaching the plateau value that should occur in the long-time limit. Therefore, in small systems it is easier to find the unjammed configuration while in large systems the zero energy states are not reached due to the larger configuration space. As a consequence of the system size analysis one can conclude, that if too small systems were considered, one would observe a ergodicity breaking only at larger densities. For example, in the limit $p \to 0$ the apparent packing fraction of the ergodic to non-ergodic transition would be much larger than 0.55 if systems with 10^3 to 10^4 particles were considered as it is the case in many simulations of glassy dynamics.

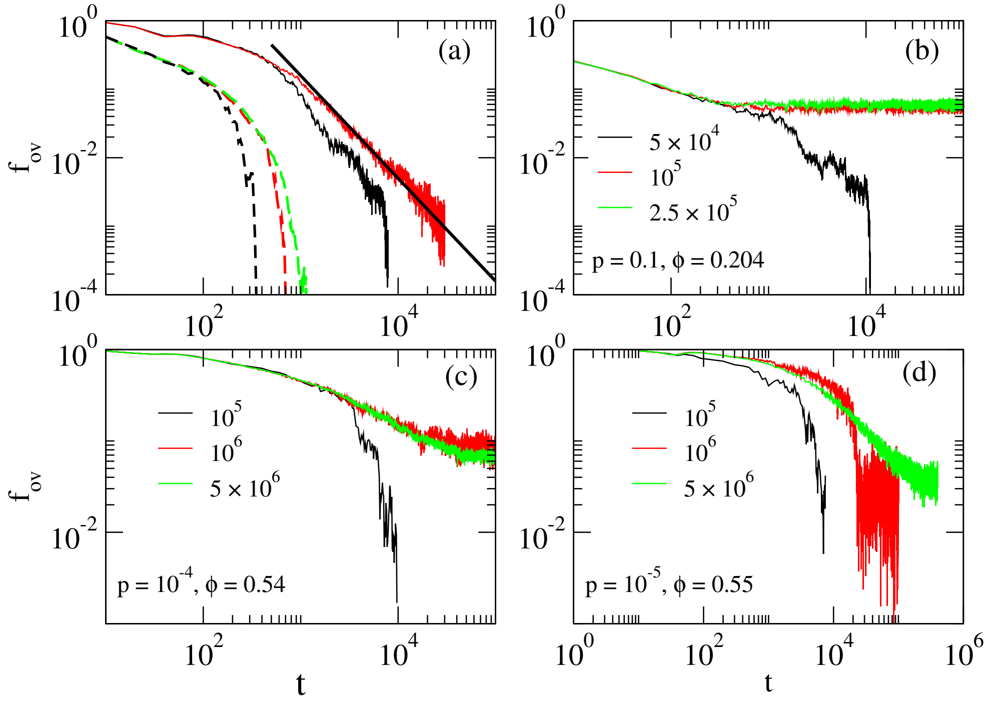

Supplementary Figure 2: System size dependence. Analysis of system size effects close to the thermal jamming transition for the relaxation process given by the fraction of overlapping particles $f_{\mathrm{ov}}(t) = N_{\mathrm{ov}}(t)/N$ as a function of step number t. (a) Typical finite size effects below the transition packing fraction for $p = 0.01$ (dashed lines) and for $p = 10^{-5}$ (solid lines). For $p = 0.01$ the packing fraction $\phi = 0.388$ just below the transition $\phi_c = 0.3998$ (as determined by a fit to the critical power law) is used for three system sizes $N = 10^4$ (black), 10^5 (red), and $N = 5 \times 10^5$ (green). For $p = 10^{-5}$ we show data for $\phi = 0.52$ (which is below the transition at $\phi_c = 0.553$) for $N = 10^6$ (black) and $N = 10^7$ (red). The black line indicates a power law with an exponent -1.5 shown for comparison. (b,c) Typical finite size effects above the transition packing fraction: (b) $p = 0.1$ and $\phi = 0.204$ (transition at $\phi_{\mathrm{c}} = 0.199$ determined by a fit to the critical power law) and (c) $p = 10^{-4}$ and $\phi = 0.54$ ($\phi_c = 0.5398$). (d) Finite size effects close to the transition for $p = 10^{-5}$ and $\phi = 0.55$.

In Fig. 2(d) we also show a case for $p = 10^{-5}$ where an unexpected jump at intermediate system sizes occurs (red curve for $N = 10^6$), where the relaxation first seems to decay rapidly but then fluctuates around a non-zero plateau value. Such a behavior is only observed in a few cases at small p and close to the transition.

In order to determine the thermal jamming transition independent of the system size we use the following recipe: The system size for a given p at first is chosen from the criteria $Np = 1$ such that at each step one random move is performed, then we increase N until for the curves that are closest to the transition we do not observe any significant change for two different system sizes.

The system sizes that we determined by this method and that we actually used for the curves shown in Fig. 2 are $N = 50000$ for $p = 1$, $N = 10^5$ for $p = 0.1$, $N = 10^6$ for $p = 10^{-3}$ and $p = 10^{-4}$ as well as for $p = 10^{-5}$ below the transition, $N = 5 \times 10^6$ for $p = 10^{-5}$ above the transition, and $N = 8 \times 10^6$ for $p = 5 \times 10^{-6}$. In order to further narrow the ranges in which the transition occurs, we sometimes employ even larger systems in Fig. 3 and Fig. 4 with up to $N = 10^7$ particles, e.g., in case of $p = 10^{-6}$.

Note that the dependence on system size is most prominent close to the transition. As a consequence the system size in principle might limit how close we can get to the transition without being affected by finite size effects. For the resolution of ϕ that we used in our article this usually is not a problem. Only for $p > 10^{-5}$ we were not able to further narrow the range of the transition packing fraction, e.g., in Fig. 4 of the main article we cannot report any plateau values $f_{\text{ov}}(t \to \infty)$ close to 0 for $p > 10^{-5}$ because only data that has proven to be independent of the system size is shown. Furthermore, for $p \geq 10^{-5}$ we are not able to determine values of τ that do not depend on the choice of the fitting range or how the data points of the relaxation curve are spaced. The reason is that for small p we are not able to get close enough to the transition such that the range of the power law behavior that is assumed for the fitting (see also Fig. 3(a)) is too small in order to obtain a reliable fit. Therefore, there are no τ-values for $p \geq 10^{-5}$ in Fig. 4(b) but only values for $f_{\text{ov}}(t \to \infty)$ in Fig. 4(a) which is still independent of the way of fitting. If one wanted to obtain values of τ for smaller p one would need larger systems closer to the transition such that the power law behavior would be sufficiently extended.

Supplementary Note 3:
Close to the thermal jamming transition: Relaxation curves and pair correlation functions

The fraction of overlapping particles $f_{\text{ov}}(t) = N_{\text{ov}}(t)/N$ as a function of step number t decreases like a power law t^{α} close to the thermal jamming transition. Close to the transition, the relaxation curves can be fitted with the function given in Eq.(2) where we assumed $\alpha = \frac{\beta}{\nu} = -0.732$ as for directed percolation. The quality of this choice of functional behavior is demonstrated in Fig. 3(a) where we collapse our data for the relaxation curves with different p and for ϕ just above the transition by plotting $F = \tau^{0.732}(f_{\text{ov}} - f_{\text{ov}}(t \to \infty))/A$ as a function of $\frac{t}{\tau}$. All curves are in good agreement with $(t/\tau)^{-0.732} \exp(-t/\tau)$ (magenta line) as expected from Eq.(2).

In Figs. 3(b) and (c) we plot the pair correlation function $g(r)$ for thermally jammed structures with ϕ just above the thermal jamming transition and for various p. In case of the non-overlapping part shown in Fig. 3(c), between the contact distance and the first minima of $g(r)$ we observe

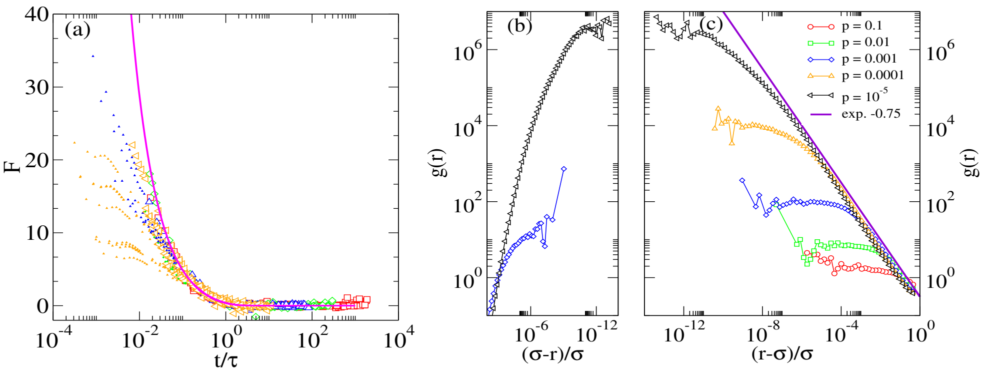

Supplementary Figure 3: Relaxation curve and pair correlation function. (a) Collapse of relaxation functions to demonstrate the agreement with Eq.(2). $F = \tau^{0.732}(f_{\text{ov}} - f_{\text{ov}}(t \to \infty))/A$ as a function of $\frac{t}{\tau}$ is plotted for different p for cases close to the transition. The colors are the same as in (c). All curves collapse onto $(t/\tau)^{-0.732} \exp(-t/\tau)$ (magenta line) as expected from Eq.(2). (b) Log-log-plot of the pair correlation function $g(r)$ shown for distances $\sigma - r$, i.e., for small overlaps. (c) Log-log-plot of the pair correlation function $g(r)$ shown for distances $r - \sigma$, i.e., for the cases where particles are close to contact but do not touch. The curves (b) and (c) are shown for structures obtained at packing fractions just above the thermal jamming transition. Before the first minimum in (c), all curves decay like a power law with exponent -0.75 (magenta line).

a power law decay with an exponent -0.75. The exponent is robust for all p. For $p \to 0$ the power law extends closer to the contact point. Note that for athermal jamming a power law of $g(r)$ is also observed[S2]. However, in case of athermal jamming the exponent is -0.5[S2] which significantly differs from the exponent -0.75 that we observe here for all values of p. Therefore not only the nature of the thermal jamming transition is fundamentally different from the athermal jamming transition as demonstrated in the main text but also for the structural properties it makes a huge difference whether a system is athermally jammed or thermally jammed.

Supplementary References

[S1] Milz, L. and Schmiedeberg, M. Connecting the random organization transition and jamming within a unifying model system. *Phys. Rev. E* **88**, 062308 (2013).

[S2] Silbert, L. E., Liu, A. J., and Nagel, S. R. Structural signatures of the unjamming transition at zero temperature. *Phys. Rev. E* **73**, 041304 (2006).
